# Fabrication of Mo-Doped WO_3_ Nanorod Arrays on FTO Substrate with Enhanced Electrochromic Properties

**DOI:** 10.3390/ma11091627

**Published:** 2018-09-05

**Authors:** Bao Wang, Wenkuan Man, Haiyang Yu, Yang Li, Feng Zheng

**Affiliations:** 1 The State Key Laboratory of Refractories and Metallurgy, Key Laboratory for Ferrous Metallurgy and Resources Utilization of Ministry of Education, Wuhan University of Science and Technology, Wuhan 430081, China; wangbao1983@wust.edu.cn; 2 School of Metallurgical and Ecological Engineering, University of Science and Technology Beijing, Beijing 100083, China; kevinman1990@163.com (W.M.); yuhaiyang0218@gmail.com (H.Y.); 3 Nano-Science and Nano-Technology Research Center, Materials Science and Engineering College, Shanghai University, Shanghai 200444, China

**Keywords:** WO_3_, Mo doping, nanorods array, hydrothermal method, electrochromic

## Abstract

Well-oriented and crystalline WO_3_ nanorod arrays (WNRAs) decorated with Mo were synthesized on fluorine doped tin oxide (FTO) substrate by the hydrothermal method. The effects of Mo doping, hydrothermal reaction time, and hydrothermal temperature on the morphologies and electrochromic properties of as-prepared WNRAs were studied thoroughly. Scanning electron microscopy (SEM), energy dispersive spectrometry (EDS), X-ray diffraction (XRD), X-ray photoelectron spectroscopy (XPS), and chronoamperometry techniques were used to characterize the structures and properties of obtained WNRAs. The results demonstrate that the average diameter of the as-prepared WNRAs ranged from 30 to 70 nm. During the decoration of Mo on the WNRAs, the growth density of as-prepared WNRAs decreased and the surfaces became rough. However, the decorated Mo on WNRAs synthesized at 180 °C for 5 h with a Mo/W mole ratio of 1:40 exhibited better electrochromic properties than single WNRAs. They exhibited high optical modulation (61.7%), fast bleaching/coloring response times (3 s/9 s), high coloration efficiency values (73.1 cm^2^/C), and good cycling stability.

## 1. Introduction

At present, tungsten trioxide (WO_3_) is a multipurpose usage material on account of its polyvalence and varied crystal forms. It has shown fascinating potential in the application of various gas sensors [1,2,3], photo catalysis [4,5], solar cells [6,7], lithium ion batteries [8], supercapacitors [9,10], and electrochromic (EC) devices [11,12]. WO_3_ is well known for its good charge storage/transfer properties and non-stoichiometric properties, as the lattice can withstand many oxygen vacancies. This characteristic feature makes it capable of exhibiting an excellent electrochromic activity by applying a small voltage across the film.

The WO_3_ film was deposited on the electrochromic devices by sputtering, which creates a uniform structure, but with a high cost of nearly $1000 per/m^2^ of glass [12,13]. Hence, intensive studies have been carried out to prepare WO_3_ thin film on transparent conductive substrates by a simple and inexpensive method. Recently, a few researchers [14,15,16,17] synthesized monoclinic WO_3_ nanowire arrays by the chemical vapor deposition (CVD) process. Li et al. [18] demonstrated the successful preparation of WO_3_ nanosheets by a novel and facile synthetic method, and then obtained 3D quasi-vertical nanosheet architectures. Zheng et al. [19] synthesized orientation-controlled h-WO_3_ nanostructures on indium tin oxide (ITO) substrates on a large scale via a simple hydrothermal method. In contrast to the sputtering approach, which requires sophisticated equipment and rigorous conditions, synthetic routes, especially hydrothermal methods, are more appropriate, controllable, and cost-effective for producing large-scale, well-ordered WO_3_ nanowires or nanorod arrays.

As commercially viable electrochromic devices, electrode materials with long-time cyclic stability, good corrosion resistance, high coloration efficiency, fast switching speed, large optical modulation, and high transmittance are the most important parameters. Crystalline WO_3_ nanorod arrays (WNRAs) synthesized on transparent substrates by the hydrothermal method offer an excellent quality, well-ordered, and uniform structure; multiple oxidation states; as well as a high capability to accommodate intercalated positive ions [20]. However, in practice, a poor coloration efficiency (~68 cm^2^/C) and long switching time (~27 s) are normally yielded by pure crystalline WO_3_ and these difficulties have not been resolved thoroughly [21]. Hence, the use of EC devices in commercially viable applications requires further improvement on their electrochromic characteristics (reversibility, stability, optical modulation, etc.). Numerous methodologies have been assumed to modify the WO_3_ film based on electrochromic devices to increase the coloration efficiency and cyclic stability. Man et al. [22] synthesized WO_3_ nanorod arrays on fluorine-doped tin oxide (FTO) substrate and precoated with a layer of TiO_2_ seeds. The results showed that WO_3_ nanorod arrays synthesized on the TiO_2_ seed layer exhibited higher coloration efficiency (142.7 cm^2^/C) and larger optical modulation (77.5%, at 660 nm) compared with those grown on the WO_3_ seed layer. Kirchgeorg et al. [23] developed electrochromic electrodes based on amorphous binary oxides, such as W−Ta oxide, with enhanced cyclic stability in the acidic electrolytes under a range of coloration voltages as low as −0.3 V. It is indicated that superior properties can be accomplished by using a mixture of two electrochromic oxides, which possess different desired properties, resulting in the obtained films holding a combination of the good characteristics [24]. Zhou et al. [25] noted that by adding Mo as a second element on WO_3_ nanorods, the performance of as-prepared gas sensors might be improved. Song et al. [26] found that the doping of Mo enhanced the photo activity of prepared WO_3_ nanowires. These studies prove that the doping of Mo on WO_3_ film produces better optical properties. However, there have been few described reports about the influence on the electrochromic performance of Mo-doped WO_3_ film. Moreover, the electrochromic properties of WNRAs with triphasic composite nanostructure (TiO_2_-MoO_3_-WO_3_) were rarely investigated [13,27].

In the present discussion, Mo-doped WO_3_ nanorod array films were synthesized by a hydrothermal method on FTO-coated glass substrates, which was pre-coated with a TiO_2_ seed layer in accordance with our previous research [22]. The effects of the Mo doping amount, hydrothermal temperature, and time on the morphology, structure, and electrochromic properties of as-prepared Mo-doped WNRAs films were systematically investigated. The cycling stability, optical modulation, response time, and coloration efficiency of WNRAs films obtained under different conditions were studied in detail. The main goal of the present study was to analyze the relationship between the morphology and electrochromic properties of mixed Mo/W oxide films further optimizes the electrochromic performance of this composite oxide material.

## 2. Experimental

### 2.1. Materials

All the reactants and solvents were of analytical grade and used without further purification. The fluorine-doped tin oxide (FTO, 10–14 Ω cm^−2^, Lanbo Glass Co., Ltd., Shenzhen, China) conductive glass was used as the substrate and shaped into a rectangular shape with dimensions of 1 × 4 cm^2^. Prior to the experiments, the FTO substrates were sonicated in acetone and ethyl alcohol (for 10 min each) followed by rinsing with deionized water, and then they were dried in a nitrogen stream.

### 2.2. Substrate Pre-Treatment

According to an as-reported method [22], the FTO substrates were pre-coated with a TiO_2_ seed layer by spin-coating using TiO_2_ colloid solution and then annealed in air. In brief, tetrabutyl titanate (TBT, China National Pharmaceutical Group Corporation, Beijing, China), as the initial reactant, was first dissolved in ethanol and is denoted as Solution A. Then, ethanol, deionized water, and hydrochloric acid (37% HCl, China National Pharmaceutical Group Corporation, Beijing, China) were mixed homogeneously to form Solution B. Subsequently, the two solutions were mixed together in a dropwise manner of Solution B into Solution A, followed by continuous stirring for 24 h at room temperature to yield a homogeneous and stable colloid solution. The final composition of the TiO_2_ colloid in a molar ratio was TBT/H_2_O/HCl/ethanol = 1:1.2:0.15:20. After that, the obtained TiO_2_ colloid solution was dropped onto the conductive side of the FTO substrate by spin-coating (KW-4A, made by Institute of Microelectronics of Chinese Academy of Science, Beijing, China) at a speed of 3000 rpm for 30 s. Finally, the as-coated FTO substrates were annealed at 700 °C for 15 min to synthesize the TiO_2_ seed layer, the thickness of which is about 200 nm [22].

### 2.3. Fabrication of Mo-Doped WNRAs on FTO Substrate

Based on previous reports [22,28], the precursor solutions were prepared for the fabrication of WNRAs as follows. Sodium molybdate dihydrate powder (Na_2_MoO_4_·2H_2_O) and sodium tungstate dihydrate powder (Na_2_WO_4_·2H_2_O) were mixed in different molar ratios R_Mo/W_ = 0 (0: 8.25 g), R_Mo/W_ = 1:40 (0.147 g: 8.04 g), and R_Mo/W_ = 1:20 (0.288 g: 7.85 g) and were dissolved in 25 mL of deionized distilled water (DDW). After that, the pH values of the precursor were changed and subsequently adjusted by the following steps. Initially, the precursor solution was acidified to pH 2.0 with the HCl solution (2 mol/L) using magnetic stirring, and a white precipitate was produced. In a further step, the solution was diluted to 250 mL by adding DDW, and oxalic acid (H_2_C_2_O_4_) was poured into this mixture solution to adjust the pH value to 2.0. Finally, a homogeneous precursor solution was obtained after stirring the solution for 30 min. To achieve hydrothermal growth, 30 mL of the obtained precursor solutions were moved into a 50 mL Teflon-lined autoclave, and then rubidium sulfate (Rb_2_SO_4_, 0.0025 mol) was added to it. The pre-treated FTO substrates were placed in autoclaves with the TiO_2_ seed layers facedown. After that, the filled autoclaves were wrapped and positioned in an oven with the temperature reaching a preset value and holding for the required amount of time to achieve a hydrothermal reaction. After the reaction, the autoclaves were cooled down to room temperature naturally. Consequently, the as-prepared substrates were rinsed repeatedly with deionized water and dried in air for further characterization.

### 2.4. Characterization and Analysis Methods

The surface morphological structure and shape of the as-prepared nanostructured WO_3_ film was characterized by a field emission scanning electron microscope (FESEM, Zeiss Supra-55, Zeiss, Jena, Germany) and a high-resolution transmission electron microscope (HRTEM, Tecnai F20, FEI, Hillsboro, USA). The phase structure was investigated by X-ray diffraction using Cu Kα radiation (λ = 0.154056 nm) with 40 kV, 200 mA, and a scan speed of 10°/min (XRD, Bruker AXS, D8, Bruker, Karlsruh, Germany). The valences of transition elements were analyzed by X-ray photoelectron spectroscopy (XPS, Al Kα, AXIS ULTRA, Shimadzu, Tokyo, Japan), in which C1s binding energy value (284.8 eV) was elected as standard for baseline correction and the baseline subtraction method (Shirley-type). Electrochemical property measurements were performed using a three-electrode system on an electrochemical workstation (CHI660E, Shanghai Chenhua Instrument, Inc., Shanghai, China). The Mo-decorated WNRAs, an Ag/AgCl electrode, and a Pt plate were used as the working, reference, and counter electrodes, respectively. Cyclic voltammetry (CV) measurements were performed in a beaker with 80 mL (1 mol L^−1^ LiClO_4_ + propylene carbonate (PC)) electrolyte between −1.0 and 1.0 V at a scan rate of 100 mV s^−1^. Chronoamperometry of the WO_3_ films was carried out by applying a voltage of ±3.0 V for 100 s in a colorimetric cell (1.0 cm × 4.2 cm) with 3 mL (1 mol/L LiClO_4_) of electrolyte. A UV-vis spectrophotometer (TU-1901, Beijing Purkinje General Instrument Co., Ltd., Beijing, China) was used to measure the transmittance spectra in the spectral region between 360 and 800 nm. The in situ coloration/bleaching switching characteristics were recorded with a UV-vis spectrophotometer with a wavelength of 600 nm.

## 3. Results and Discussion

### 3.1. Effect of Mo Doping on the as Prepared WNRAs Films

**Figure 1:**
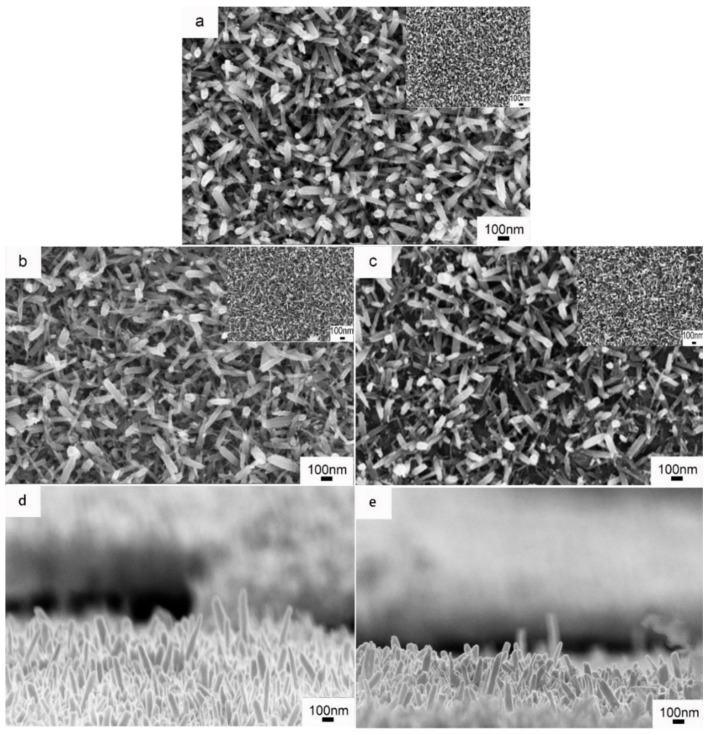
Scanning electron microscopy (SEM) images of WO_3_ nanorod arrays (WNRAs) film prepared with different molar ratios of Mo/W (R_Mo/W_) (**a**) 0; (**b**,**d**) 1: 40; (**c**,**e**) 1: 20.

Various molar ratios of Mo/W (R_Mo/W_) were taken to control the structure of the film. In detail, the Mo/W ratio was adjusted to 0, 1:40, and 1:20 to investigate the influence of Mo doping on the microstructure and electrochromic properties of as-prepared WNRAs films. The acquired WNRAs films were labeled WNRAs-0, WNRAs-1, and WNRAs-2, respectively. Figure 1 shows the SEM images of the as prepared WNRAs films at 180 °C for 4 h with different R_Mo/W_. The results indicate that the accomplished film was composed of nanorods without noticeable differences in morphology but the average diameter and length of the nanorods were diminished with the increase of R_Mo/W_. It can be seen that the average diameter of as-prepared nanorods decreased from 44.7 to 37.4 nm with the increase of Mo concentration from 1:40 to 1:20. Meanwhile, the average length of obtained nanorods decreased from 568 to 473 nm, too. In addition, the density of the as-prepared nanorod arrays was reduced simultaneously, demonstrating the doping of Mo has some influence on the growth of WNRAs. It could be ascribed that the structures of Mo^6+^ and W^6+^ ions are very similar, and Mo^6+^ ions may be attached to the structure of WO_3_, which defeats the growth of formed WO_3_ nanorods [29]. Furthermore, MoO_3_ exhibited better kinetic characteristics compared with WO_3_ at a higher growth rate at lower hydrothermal temperatures [14]. Hence, MoO_3_ crystal nuclei may be formed on the substrate, especially at relatively low temperatures and occupied some sites for the formation of WO_3_ nanorods, leading to a decrease in the nanorod array density of the prepared WO_3_.

**Figure 2:**
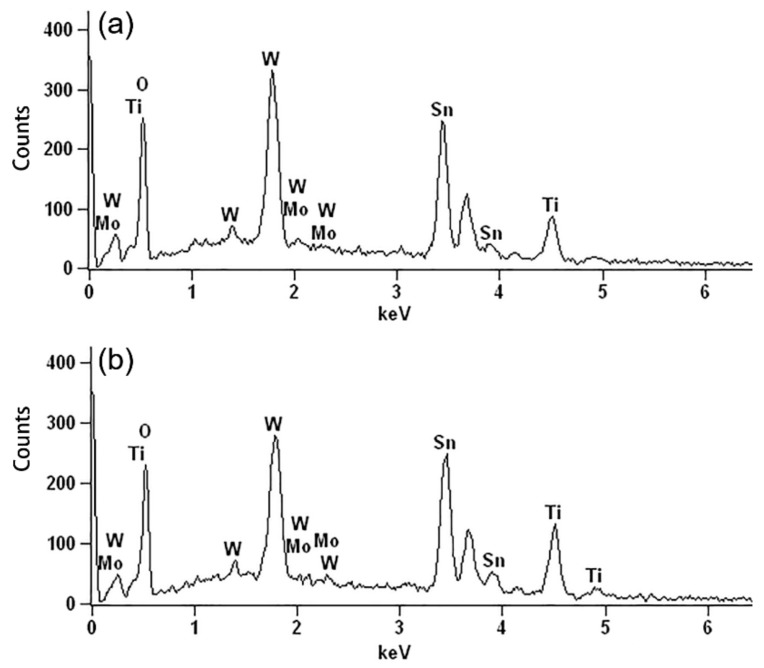
Energy dispersive spectrometry (EDS) spectra of prepared WNRAs film with different R_Mo/W_ (**a**) 1:40; (**b**) 1:20.

**Figure 3:**
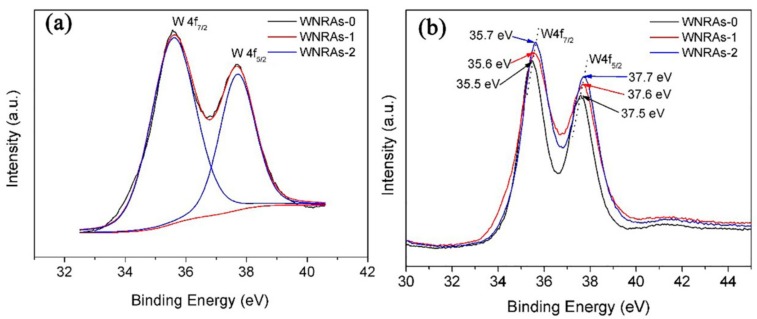
X-ray photoelectron spectroscopy (XPS) spectra of as-prepared WNRAs film with different R_Mo/W_ (**a**) 1:40; (**b**) different R_Mo/W_.

In addition, the energy dispersive spectrometry (EDS, Zeiss, Jena, Germany) spectra shown in Figure 2 indicate that Mo certainly existed in the prepared WNRAs film. Meanwhile, the peak intensity of W was much stronger than that of Mo, suggesting that WO_3_ dominated and the amount of Mo doped in WO_3_ was very small. The atomic concentrations of Mo in the obtained films are about 0.47% for WNRAs-1 and 0.96% for WNRAs-2 from the EDS spectra. To confirm that Mo was doped into the structure of the as-prepared WO_3_ nanorod, XPS measurements were performed to detect the chemical composition and valance state of WO_3_ prepared with different precursors. In the W4f core level spectrum shown in Figure 3a, the binding energies of the double peaks were 35.6 and 37.6 eV for W4f_7/2_ and W4f_5/2_, respectively [30,31,32]. The energy position of this doublet corresponds to the W^6+^ oxidation state, indicating that the valance state of W is still +6 with the doping of Mo. However, when the R_Mo/W_ of precursor increased from 1:40 to 1:20, the well-resolved split doublet peaks corresponding to W4f_7/2_ and W4f_5/2_ shifted to 35.7 and 37.7 eV, respectively (Figure 3b). This might be ascribed to the doping of Mo, leading to the shift of binding energy of WO_3_ [31,32,33,34].

**Figure 4:**
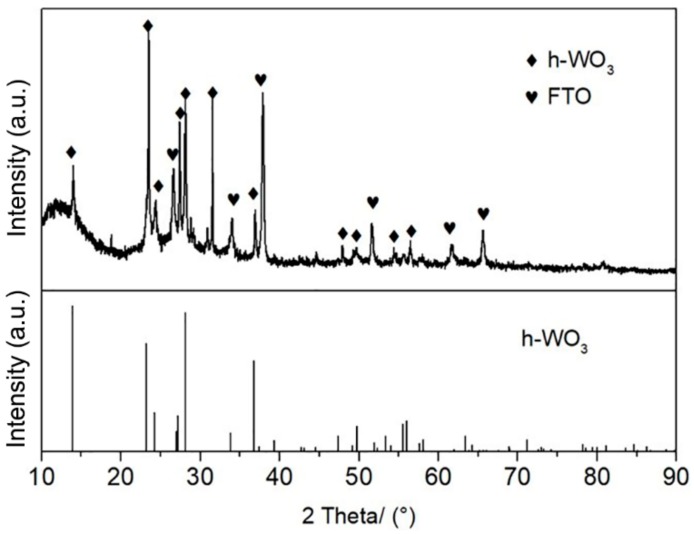
XRD pattern of as-prepared WNRAs film at 180 °C for 4 h with R_Mo/W_ of 1:40. FTO—fluorine doped tin oxide.

More detailed morphological and structural features of WNRAs film synthesized at 180 °C for 4 h with a R_Mo/W_ of 1:40 were studied by XRD and TEM. Figure 4 is the XRD of as-prepared WNRAs film. All the diffraction peaks are indexed to hexagonal WO_3_ (JCPDS 01-085-2460) except for the substrate of FTO, which indicate that the crystalline phase of obtained WNRAs is WO_3_ with a hexagonal phase (h-WO_3_).

**Figure 5:**
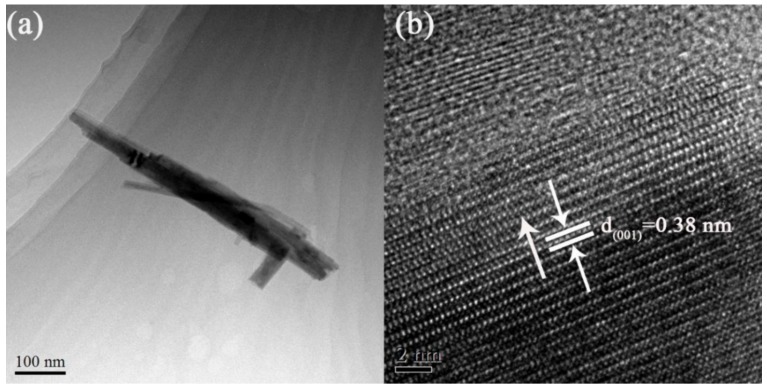
Transmission electron microscope (TEM) and high-resolution transmission electron microscope (HRTEM) images of WNRAs prepared at 180 °C for 4 h with R_Mo/W_ of 1:40 (**a**) TEM; (**b**) HRTEM.

As the TEM image shows in Figure 5a, the obtained nanorods had a needle-like structure, an average diameter of 30–70 nm, and length of 550 nm. Moreover, it can be seen from the high-resolution (HR)TEM image (Figure 5b) that the lattice interplanar spacing was about 0.38 nm, which corresponds well to the (001) lattice plane of the hexagonal structure of WO_3_, indicating that the prepared WO_3_ nanorods grow along the (001) direction. It agrees well with the results shown in other literature [35,36,37], suggesting that the doping of Mo does not significantly change the growth orientation of synthesized WO_3_ nanorods.

**Figure 6:**
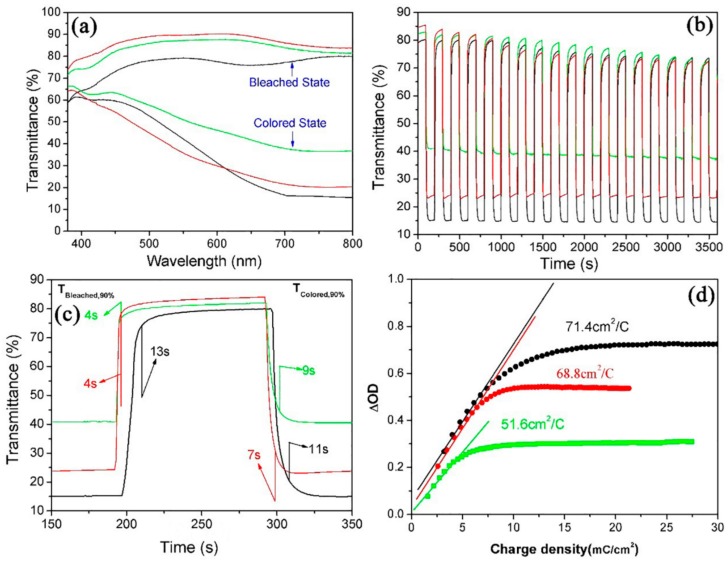
Electrochromic properties of WNRAs prepared with different R_Mo/W_ (black line: 0, red line: 1:40, green line: 1:20) (**a**) UV-vis transmittance spectra in colored and bleached states measured at ±3.0 V for 100 s; (**b**,**c**) switching time characteristics between the colored and bleached states measured at ±3.0 V with a wavelength of 660 nm; (**d**) coloration efficiency at 660 nm.

It is well known that the structure and morphology of prepared WO_3_ film play important roles in determining its electrochromic properties. Herein, the electrochromic performances of WNRAs obtained with different precursor ratios were investigated. The maximum optical modulation was one of parameters measured to elucidate the electrochromic properties of WO_3_ film, which is defined as the maximum difference between the transmittance of WO_3_ film at colored and bleached states in the visible region. Figure 6 explains the electrochromic properties of WNRAs obtained in different molar ratios of W to Mo in precursor. It can be clearly seen from Figure 6a that the maximum optical modulation of coloration/bleaching at 660 nm is about 55.5% for WNRAs-0. Compared with WNRAs-0, WNRAs-1 was shown to offer much larger transmittance in the bleached state (88.6%) and similar transmittance in the colored state (24.2%) at a wavelength of 660 nm, leading to a larger optical modulation (64.4%). This is ascribed to the decrease in the density of the as-prepared nanorod array with the doping of Mo, leading to an increase in transmittance in the bleached state. However, with an increase in the doping amount of Mo, the maximum optical modulation of coloration/bleaching decreased to 45.2%. This is because excessive doping of Mo would destroy the structure of prepared WNRAs and result in poorer crystallization of obtained sample [26], which would lead to lower optical modulation of WNRAs-2 than that of WNRAs-1.

For the switching characteristics of as-prepared WNRAs in different precursors, in situ transmittance curves at 660 nm of these samples are shown in Figure 6b,c. From Figure 6b, it can be seen that the transmittance of these samples did not lead to a big change in the colored states, while a small decrease of the transmittance occurred in the bleached state after a series of cycles. This is attributed to Li^+^ ions still being stuck in the WO_3_ nanorods during the charge and discharge processes. The switching time was defined as the time required for a 90% change in the full transmittance modulation at a certain wavelength [13,22]. As shown in Figure 6c, the switching times of WNRAs-0 at 660 nm for coloring and bleaching were 13 and 11 s, respectively. However, as for WNRAs-1 and WNRAs-2, the coloration time was 4 s and the bleaching time was about 7−9 s, which are much faster than that of WNRAs-0. This indicates that the Li-ions and electrons are easier to intercalate or deintercalate to the WO_3_ crystal lattice with the existence of Mo doping.

This phenomenon can be explained by the following two features: initially, with the doping of Mo on WO_3_ nanorods, the density and average diameter of the as prepared WO_3_ nanorods were decreased, which allowed the electrolyte to contact the WO_3_ nanorods. Moreover, the smaller diameter shortened the diffusion distance of Li-ions in nanorods, which was conducive to the intercalation or deintercalation of Li-ions. Another aspect was that the doping of Mo on the WO_3_ nanostructure would have resulted in the distortion of WO_3_ crystal, as the diameters of these ions were different (Mo^6+^ = 0.055 nm, W^6+^ = 0.056 nm), which would generate more and wider channels for the Li-ions and electrons to intercalate into and deintercalate out of the WO_3_ crystal lattice.

In addition, another key factor in the determination of electrochromic properties is the coloration efficiency (CE) of WO_3_, which is defined as the change in optical density (OD) per unit of charge density (Q/A) during switching. It can be calculated according to the formula: CE = ∆OD/(Q/A) [38,39], where OD = log(T_b_/T_c_). T_b_ and T_c_ refer to the transmittance of the film in its bleached and colored states, respectively. Figure 6d shows the plots of OD at a wavelength of 660 nm vs. the charge density at a potential of −3.0 V. The coloration efficiency was obtained from the slope of the lines fitted to the linear region of the curves. It was indicated that the coloration efficiency of WNRAs-0 is 71.4 cm^2^/C. However, with the doping of Mo in WO_3_, the coloration efficiency of WNRAs-1 and WNRAs-2 deceased to 68.8 cm^2^/C and 51.6 cm^2^/C, respectively. This can be ascribed to the termination of the crystal structure and diminution of the conductivity of WO_3_ by doping of Mo on WO_3_, which are disadvantages for the diffusion of Li-ions and electrons in prepared WNRAs, resulting in the decrease of coloration efficiency in the obtained samples.

**Figure 7:**
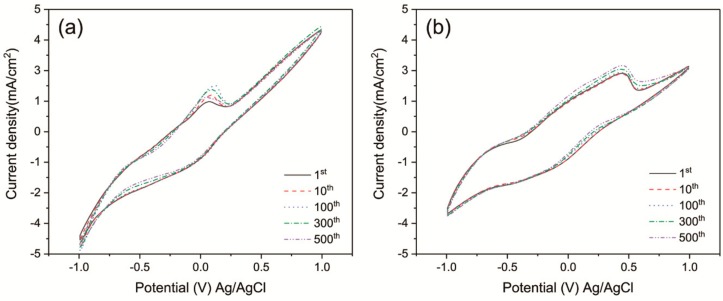
Cyclic voltammetry curves of (**a**)WNRAs-1 and (**b**) WNRAs-2 at different cycles at the scanning rate of 100 mV/s.

In order to further investigate the cycling stability of obtained film, the cyclic voltammograms (CVs) are used to elucidate the long-term durability of WNRAs-1 and WNRAs-2, respectively. As shown in Figure 7, there is no significant change in the shape of CV curves after 500 cycles, indicating that the as-prepared WNRAs have good long-term durability. Moreover, the ions intercalation/deintercalation capacities of the film could be calculated by integrating the CV curves. When the prepared films cycled for 10 cycles, the ratios of ions deintercalation/intercalation is about 92.0% for WNRAs-1 and 89.8% for WNRAs-2, respectively, indicating that there were still Li^+^ ions left in the films at the bleaching state.

Based on the aforementioned analysis, it is known that the doping of Mo has some effects on the morphology of prepared WNRAs and further influences the electrochromic properties of obtained WNRAs obviously. WNRAs-1 presented larger optical modulation and a faster coloration time compared to WNRAs-0 and WNRAs-2, indicating that the doping of Mo in a suitable amount has the advantage of superior electrochromic properties.

### 3.2. Effect of Hydrothermal Time on Microstructure and Electrochromic Properties of as-Prepared WNRAs Films

**Figure 8:**
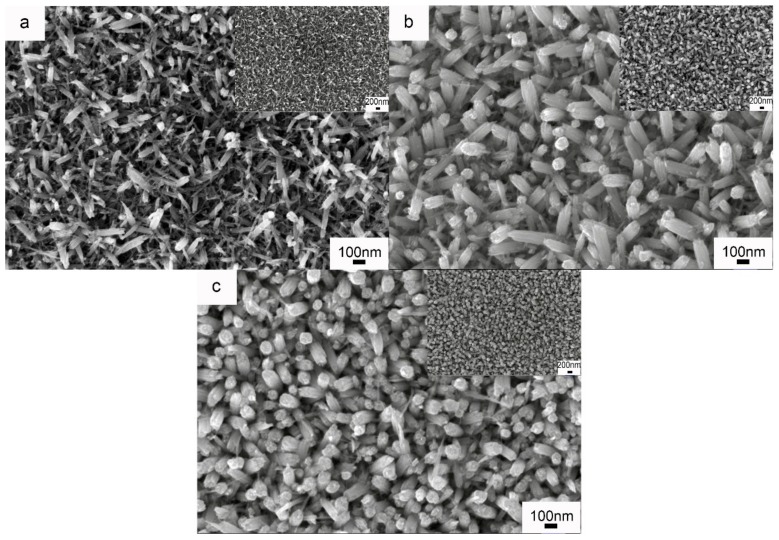
SEM images of prepared WNRAs at 180 °C for different time (**a**) 4 h; (**b**) 5 h; (**c**) 6 h.

Because the WNRAs-1 synthesized in the precursor with an R_Mo/W_ of 1:40 exhibited better electrochromic properties, the investigations into the influence of the hydrothermal time on the WNRAs films were conducted at a fixed temperature of 180 °C and an R_Mo/W_ of 1:40. Figure 8 shows the morphologies of prepared WNRAs at 180 °C for various time intervals. The hydrothermal time ranged from 4 to 6 h and the obtained samples were named WNRAs-4 h, WNRAs-5 h, and WNRAs-6 h. It was found that the average diameters of the obtained nanorods increased obviously with the prolonging of hydrothermal time, which, in turn, resulted in an increase in the density of as-prepared WNRAs. Moreover, the nanowire distributed in the gaps of nanorods disappeared as the hydrothermal time was prolonged from 4 to 6 h, indicating that the increase in the hydrothermal time was beneficial for the conversion from nanowires to nanorods.

**Figure 9:**
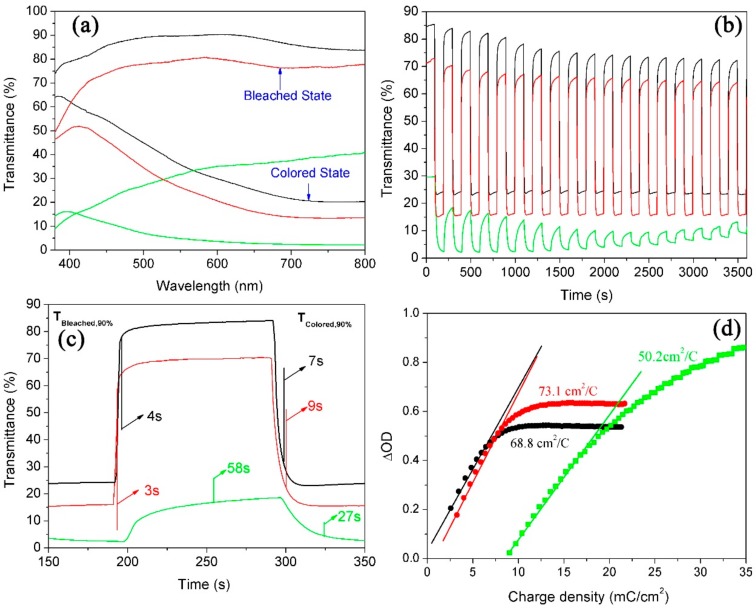
Electrochromic properties of WNRAs prepared at 180 °C for different times (black line: 4 h, red line: 5 h, green line: 6 h) (**a**) UV-vis transmittance spectra in colored and bleached states measured at ±3.0 V for 100 s; (**b**,**c**) switching time characteristics between the colored and bleached states measured at ±3.0 V with a wavelength of 660 nm; (**d**) coloration efficiency at 660 nm.

Figure 9 illustrates the electrochromic properties of WNRAs films obtained at various time intervals. As the average diameter and density of WNRAs-5 h were larger than those of WNRAs-4 h, the WNRAs-5 h presented lower transmittance in the bleached state (77.2%) and colored state (15.5%), leading to a larger optical modulation of 61.7% at a wavelength of 660 nm. Moreover, the WNRAs-6 h exhibited much lower transmittance in the bleached state and colored state only 36.2% and 2.8%, respectively. Hence, the maximum optical modulation of coloration/bleaching at 660 nm was only about 33.4% for WNRAs-6 h (Figure 9a). In addition, the cyclic reversibility and the switching time were similar with WNRAs-4 h and WNRAs-5 h, except for the coloration time, which increased slightly from 7 to 9 s. However, compared with WNRAs-5 h, the cyclic reversibility of WNRAs-6 h was much poorer, while the coloration/bleaching time was prolonged from 3 s/9 s to 58 s/27 s, respectively. (Figure 9b,c).

This phenomenon can be explained as follows: generally, the coloration process comprises the ion diffusion process and the interface reaction process. Owing to the driving force from the concentration gradients of Li-ions and the electric field, the Li-ions initially migrate to the surface of the WO_3_ nanorods from the electrolyte. Then, the Li-ions are injected into the WO_3_ crystal lattice by the force of free diffusion or the electric field. Meanwhile, the electrons transfer to the WO_3_ crystal lattice and combine with the Li-ions to form LiWO_3_ through the conductive substrate. The discoloration and coloration processes are reversible. The whole processes can be illustrated by the 

WO_3_ (transparent) + xe^−^ + xLi^+^ ⟷ 4Li_x_WO_3_ (blue). (1)

The WNRAs-5 h showed better crystallinity than WNRAs-4h. This is beneficial for Li-ions’ injection into or dejection out of the WO_3_ lattice, which is the reason for the faster coloring response of WNRAs-5 h. However, with a further increase in the density and average diameter of prepared WNRAs, the migration of Li-ions to the surface of WO_3_ nanorods is harder and the diffusion length for Li-ions in each single nanorod is longer, which retards the ion diffusion process. So, for WNRAs-6 h, the response time was much longer compared with other WNRAs.

The results are shown in Figure 9d. It is depicted that the coloration efficiencies for WNRAs-4 h and WNRAs-5 h were 68.8 and 73.1 cm^2^/C, respectively. Generally, it is supposed that a high value of coloration efficiency means that the electrochromic material exhibits large optical modulation even when a small charge is applied. Figure 9a,b verified that the prolonging of the hydrothermal reaction time from 4 to 5 h improved the electrochromic performance, which is consistent with the result shown in Figure 9d. However, the coloration efficiency for WNRAs-6 h was decreased to 50.2 cm^2^/C when the hydrothermal time increased further from 5 to 6 h, indicating that the appropriate hydrothermal time for the fabrication of WNRAs with good electrochromic properties is 5 h.

### 3.3. Effect of Hydrothermal Temperature on the Microstructure and Electrochromic Properties of As-Prepared WNRAs Films

**Figure 10:**
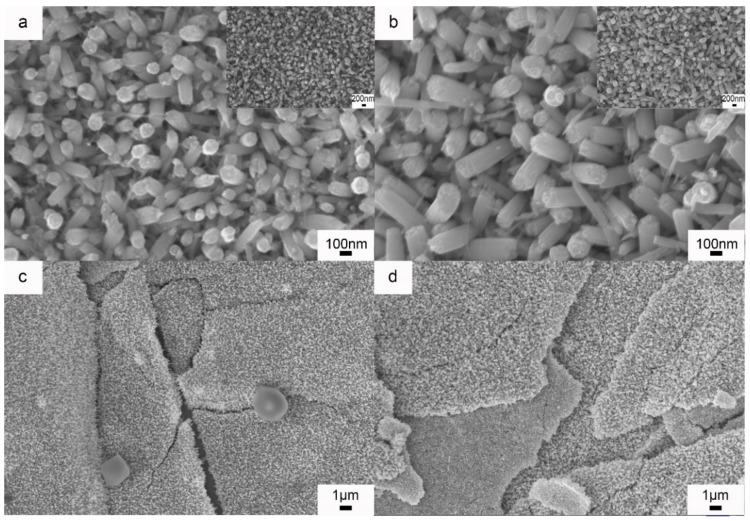
SEM images of WNRAs prepared at 190 °C for different times (**a**,**c**) 4 h; (**b**,**d**) 5 h.

To study the effect of the hydrothermal temperature on the electrochromic properties of WO_3_ nanostructures, WNRAs were synthesized at 190 °C for 4 and 5 h, respectively. Figure 10 shows the morphologies of the obtained samples. Compared with the samples prepared at 180 °C, the nanorods obtained at 190 °C for the same time showed larger diameters, indicating that higher temperatures (the same as the longer time) increased the average diameter of prepared nanorods. However, in the low-resolution images shown in Figure 10c,d, the WNRAs films overlap in some places, resulting in the formation of a layered structure. This may be because of the density of the WNRAs film in some areas being too great, leading to small pieces and staggered growth.

**Figure 11:**
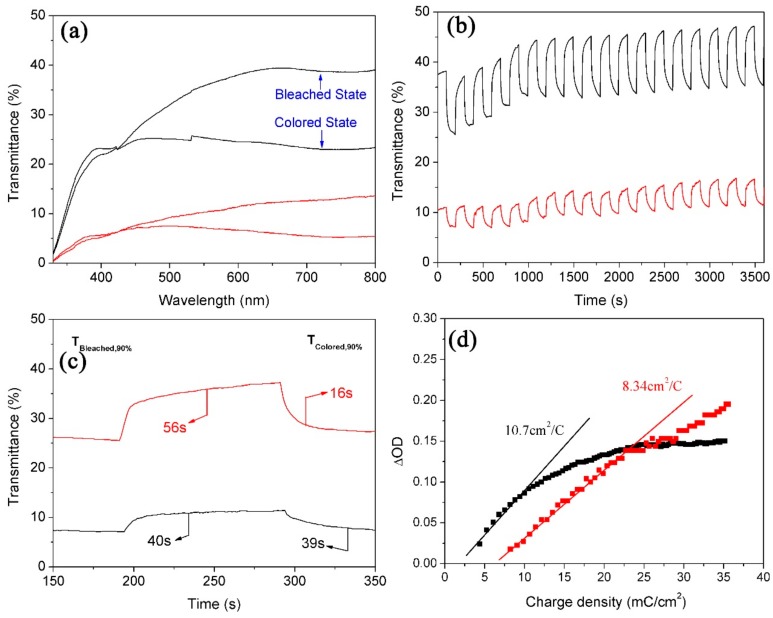
Electrochromic properties of WNRAs prepared at 190 °C for different times (black line: 4 h, red line: 5 h) (**a**) UV-vis transmittance spectra in colored and bleached states measured at ±3.0 V for 100 s; (**b**,**c**) switching time characteristics between the colored and bleached states measured at ±3.0 V with a wavelength of 660 nm; (**d**) coloration efficiency at 660 nm.

From the investigation on the electrochromic properties of WNRAs films obtained at 190 °C, it is known that their electrochromic performances are very poor. As shown in Figure 11, it was found that the transmittance of obtained WNRAs films was below 45%, no matter whether they were in the bleached state or the colored state, indicating that the as-prepared films were too thick. Moreover, the coloration/bleaching time was much longer, and the CE values were much smaller than the samples prepared at 180 °C, indicating that these WNRAs films are unsuitable for the fabrication of electrochromic devices.

The following three points show the major reasons for the poor electrochromic performance. Initially, the density of as-prepared nanorods was too large, which makes it difficult for them to contact with the electrolyte. Further, the layered structure would make it difficult for Li-ions to inject into or eject from the nanorods, which could retard the coloration/bleaching time. Last, but not the least, it is easy for films with layered structures to be exfoliated from the substrate, naturally resulting in poor cyclic reversibility and a lower CE value.

## 4. Conclusions

In summary, Mo-doped WNRAs were synthesized on FTO substrate by a simple hydrothermal method with low cost. The doping of Mo not only had some influence on the morphologies and structures of the obtained WNRA films, but also had significant effects on the electrochromic performance of the as-prepared WNRAs. The WNRAs obtained in the precursor with an R_Mo/W_ of 1:40 at 180 °C for 4 h presented larger optical modulation (64.4%) and faster coloration and bleaching times (4 s/7 s) compared with those prepared with other precursors. However, as the doping of Mo may terminate the crystal structure and decrease the conductivity of the WNRAs, the coloration efficiency of Mo-doped WNRAs is not as good as that of pure WNRAs, indicating that doping with an appropriate amount of Mo is beneficial for some specific electrochromic properties. Moreover, the effects of the hydrothermal temperature and time on the morphologies and electrochromic properties were investigated systematically. The results suggested that under optimum conditions for the preparation of WNRAs, with good morphologies and excellent properties at hydrothermal temperature of 180 °C and a growth time of 5 h, Mo-doped WNRAs with an optical modulation of 61.7% at a wavelength of 660 nm are produced with a coloration/bleaching time of 3 s/9 s and a coloration efficiency of 73.1 cm^2^/C. Especially, the coloration/bleaching time is faster than that in other literature [21,22].
